# Glycemic control and healthcare utilization following pregnancy among women with pre-existing diabetes in Navajo Nation

**DOI:** 10.1186/s12913-018-3434-x

**Published:** 2018-08-10

**Authors:** Julius Ho, Karen Bachman-Carter, Shelley Thorkelson, Kristi Anderson, Jennifer Jaggi, Chris Brown, Katrina Nelson, Cameron Curley, Caroline King, Sid Atwood, Sonya Shin

**Affiliations:** 1 Department of Medicine, Johns Hopkins School of Medicine, 1800 Orleans St, Baltimore, MD 21298 USA; 2 Northern Navajo Medical Center, Indian Health Service, Shiprock, NM USA; 3 Gallup Indian Medical Center, Indian Health Service, Gallup, NM USA; 4 Division of Global Health Equity, Brigham and Women’s Hospital, 210 East Aztec Avenue, Gallup, NM 87301 USA; 5 Community Outreach and Patient Empowerment, 210 East Aztec Avenue, Gallup, NM 87301 USA; 6 Department of Global Health and Social Medicine, Harvard Medical School, 210 East Aztec Avenue, Gallup, NM 87301 USA

**Keywords:** Native American, Pregnancy, Healthcare, Glycemic control, Post-partum, Diabetes

## Abstract

**Background:**

Native American communities experience greater burden of diabetes than the general population, including high rates of Type 2 diabetes among women of childbearing age. Diabetes in pregnancy is associated with risks to both the mother and offspring, and glycemic control surrounding the pregnancy period is of vital importance.

**Methods:**

A retrospective chart review was conducted at a major Navajo Area Indian Health Service (IHS) hospital, tracking women with pre-existing diabetes who became pregnant between 2010 and 2012. Logistic regression was performed to find patient-level predictors of our desired primary outcome—having hemoglobin A1c (HbA1c) consistently < 8% within 2 years after pregnancy. Descriptive statistics were generated for other outcomes, including glycemic control and seeking timely IHS care.

**Results:**

One hundred twenty-two pregnancies and 114 individuals were identified in the dataset. Baseline HbA1c was the only covariate which predicted our primary outcome (OR = 1.821, 95% CI = 1.184–2.801). Examining glycemic control among pregnancies with complete HbA1c data (*n* = 59), 59% were controlled before, 85% during, and 34% after pregnancy. While nearly all women received care in the immediate postpartum period, only 49% of women visited a primary care provider and 71% had HbA1c testing in the 2 years after pregnancy.

**Conclusions:**

This is the first analysis of outcomes among women with diabetes in pregnancy in Navajo Nation, the largest reservation and tribal health system in the United States. Our findings demonstrate the positive impact of specialized prenatal care in achieving glycemic control during pregnancy, while highlighting the challenges in maintaining glycemic control and continuity of healthcare after pregnancy.

## Background

Diabetes is an important area of health inequity which affects Native Americans. The prevalence of adults with diabetes is 2.4 times greater in Native Americans than whites, while the age-adjusted death rate is 1.8 times greater [1]. A range of social and historic forces have played a role in shaping this morbidity and mortality. Access to healthcare services is a challenge due to limited resources for tribal healthcare systems, chronic understaffing of providers, as well as vast geographic distances and lack of transportation [2]. On Native reservations, traditional lifestyles and food systems face pressure from a built environment that makes healthy living difficult [3].

Studies have noted the prevalence of Type 2 diabetes mellitus among young Native Americans is higher than other ethnic groups and increasing, with implications for women of childbearing age [4, 5]. Diabetes in pregnancy is a concern for multiple reasons. Physiologic changes associated with pregnancy affect insulin resistance and alter the course of diabetes management. Pre-existing diabetes in pregnancy is a major risk factor for adverse outcomes, including congenital anomalies, preterm birth, cesarean section, and overall perinatal mortality [6]. There are lifelong consequences to the offspring of mothers with diabetes, including significantly higher risk of developing metabolic syndrome and diabetes, leading to an intergenerational cycle of disease [7, 8].

Prior research has pointed to diabetes during the pregnancy period as an important driver of the epidemic among Native communities in particular [9]. One recent study of eight US states found that diabetes was reported in 5.92% of births among Native women [10]. On Navajo Nation, the last published study related to diabetes in pregnancy is nearly three decades old, and the reported prevalence rate of 4.6% (pre-existing as well as gestational) are no longer reflective of the current clinical reality or efforts to address diabetes in pregnancy [11]. Therefore, more research is needed to better understand diabetes in pregnancy outcomes in this population [12]. To address this gap, a retrospective chart review was performed of women with diabetes receiving IHS care on Navajo Nation who became pregnant. Our goal was to characterize patient-level predictors of post-pregnancy diabetes outcomes and care utilization, with the aim of identifying opportunities to improve care for this population.

## Methods

This study was a retrospective case series of a single Navajo Area Indian Health Service (IHS) facility located on Navajo Nation.

### Study context

The Navajo Nation is a sovereign nation located in the American Southwest, spanning parts of New Mexico, Arizona, and Utah. It is the largest tribal reservation in the US and contains over 170,000 inhabitants over more than 27,000 miles^2^, a rural area larger than the state of West Virginia. The Navajo Area IHS is divided into five regional service units, and operates hospitals and health centers which serve nearly a quarter-million patients living on the reservation and in surrounding communities [13]. IHS health care services are available at no cost to all enrolled members of the Navajo Nation or any other federally recognized tribe.

Both the Navajo Nation and IHS are engaged in numerous programs focusing on diabetes management [14]. Specifically for pregnant patients, there are multidisciplinary Diabetes In Pregnancy teams in each service unit which offer intensive case management, nutrition counseling, and other services on a referral basis. These teams are modeled after Sweet Success, a national standard of diabetes and reproductive health education developed by the California Diabetes and Pregnancy Program [15, 16]. The Community Outreach and Patient Empowerment (COPE) Program, a collaboration established in 2009 between Brigham and Women’s Hospital, Partners In Health, the Navajo Nation Department of Health, and Navajo Area IHS, supports community-based chronic disease management on the reservation and conducted the present analysis [17].

### Data collection

As part of an ongoing program evaluation by COPE, deidentified data were extracted from the IHS electronic health record for all patient encounters on the reservation from 2009 to 2016 with a DM Audit Problem Diabetes Diagnosis taxonomy, which includes ICD-9 and ICD-10 codes for diabetes mellitus [18]. Permission to access health data was given by the Navajo Area IHS, and this project was approved by the Navajo Nation Human Research Review Board and the Partners Healthcare Institutional Review Board.

We performed a sub-analysis including all women with a diagnosis of diabetes who were seen within a IHS particular service unit, and selected for those who had a visit for pregnancy between 2010 and 2012 to allow for adequate pre- and post-pregnancy tracking.

#### Identifying pregnancies

Pregnancies were identified by using regular expression queries to flag patients with pregnancy-related terms (related to weeks gestation, estimated due date, etc) in the narrative free-text field of their visit records between 2010 to 2012. An approximate date of conception was back-calculated from due date/gestational age at the last available visit, to obtain the most reliable estimate. Pregnancy end dates were determined by searching for the earliest visit containing an ICD-9 code for a recognized pregnancy outcome (live birth, stillbirth, spontaneous abortion, therapeutic abortion, or other) following the date of conception.

#### Covariate/exposure variable definitions

The explanatory variables in our analysis included patient characteristics such as age, body mass index (BMI), Medicaid status, trimester of first pre-natal visit, and baseline comorbid conditions.

Patient age at date of conception, BMI and baseline HbA1c were continuous variables, while Medicaid status was a binary variable defined as having ever been listed on Medicaid in the period from 1 year before to 2 years after date of conception. The trimester of first prenatal visit defined by the earliest visit to the obstetrics or gynecology clinic after date of conception: first trimester was designated as Days 1–90, second trimester was Days 91–188, and third trimester was after Day 188.

To determine which patient comorbidities to include, we referenced Practice Bulletins on pregestational and gestational diabetes published by the American College of Obstetrics and Gynecology, and consulted local clinical stakeholders. We defined the binary presence or absence of each condition using a list of ICD-9 codes adapted for the Elixhauser Comorbidity Index, a standardized method for categorizing patient comorbidities. These ICD codes were then searched for in the problem lists of patient visits in the period from 365 days prior to 180 days after the date of conception.

#### Defining healthcare utilization

Healthcare was defined as any visit that was assigned a unique internal entry number in the electronic health record. Entries produced by case management services, chart review, and telephone/mail correspondences were excluded.

We focused on care up to 8 weeks following a pregnancy end date, more flexible than the six-week postpartum period, to reflect the geographic and other barriers to care experienced by this population [19].

Healthcare utilization was assessed at three levels. We looked broadly at healthcare encounters of all types, to capture the degree of interaction patients had with the IHS system. Visits to the obstetrics, gynecology, or a primary care clinic (internal medicine, family practice, and pediatrics) were also assessed, since these settings provide pregnancy follow-up and health maintenance care. Finally, we looked at the presence of hemoglobin A1C (HbA1c) testing since it is usually sent by primary care providers as a part of routine diabetes care.

### Statistical analyses

Our primary outcome of interest was a binary composite measure. The favorable outcome was defined as having HbA1c’s consistently < 8% within 2 years after a mother’s pregnancy end date. In contrast, a case had an unfavorable outcome if she had any HbA1c measurement ≥8%, or if she had no HbA1c recorded within 2 years post-pregnancy. This threshold of HbA1c 8% was selected based on the definition of good glycemic control by IHS, which also recommends HbA1c testing at least twice yearly and more often for those who experience pregnancy per national guidelines [20, 21]. Univariate logistic regression analyses against our binary outcome were performed for each covariate using all cases with data for the variable in question.

We handled missing pregnancy outcome data by using simple imputation to presume a full-term pregnancy, and then looking at HbA1c trends from baseline pre- to post-pregnancy for all cases. For pre-pregnancy we used a wider “periconception” definition spanning from 365 days before date of conception through the first trimester, based on work reflecting the understanding that early pregnancy is categorically distinct from changes which take place in the second trimester and beyond [22, 23]. HbA1c values were considered to be post-pregnancy measures if they were collected between 280 days after the date of conception (the length of a full-term pregnancy) to 1010 days (2 years afterwards).

Looking at healthcare utilization after pregnancy, we calculated time-to-event curves using the Kaplan-Meier method for key clinical events: 1) healthcare encounter of any type, 2) visit to an obstetrics, gynecology or primary care clinic, and 3) HbA1c testing.

## Results

We identified 114 unique patients who were pregnant, out of 1749 women with diabetes of childbearing age (16 to 49 years old) who visited the facility between 2010 and 2012. When we calculated dates of conception, eight women were found to have a second pregnancy in our time window, for a total of 122 pregnancy cases included in the analysis.

Eighty-two out of 122 total cases were associated with a known pregnancy result, including 61 live births, 18 spontaneous abortions and three therapeutic abortions, from which a pregnancy end date and thus our primary clinical outcome could be determined. Of these, 25 (30%) had the favorable outcome of having measured HbA1c < 8% within 2 years post-pregnancy. The remainder of our cases had an unfavorable outcome, either an uncontrolled HbA1c (*n* = 35, 43%) or no measured HbA1c (*n* = 22, 27%). The baseline characteristics of pregnant women are presented in Table 1, in aggregate and stratified by clinical outcome. The 40 cases with unknown outcome had a significantly higher mean HbA1c than those with known outcomes (9.1% vs 7.8%, *P* = 0.021), but otherwise were similar in all other baseline characteristics (*not presented*).

**Table 1:** Baseline characteristics of pregnant women

Reported as n (%) or mean +/− SD			
Characteristics (unit)	All pregnancies, *n* = 122	Favorable outcome, *n* = 25	Unfavorable outcome, *n* = 57
Age (years)	30.8 +/− 6.2	31.9 +/− 5.8	30.2 +/− 6.1
Medicaid status	108 (89%)	22 (88%)	51 (89%)
Baseline HbA1c (%) (*n* = 90)	8.3 +/− 2.5	6.5 +/− 1.7	8.5 +/− 2.4
Baseline BMI (kg/m^2) (*n* = 71)	35.8 +/− 9.1	37.0 +/− 5.5	35.3 +/− 7.8
Baseline comorbidities			
Hypertension	31 (25%)	6 (24%)	14 (25%)
Hyperlipidemia	35 (29%)	7 (28%)	17 (30%)
Cardiovascular disease (non-hypertension)	7 (6%)	2 (8%)	3 (5%)
Depression	24 (20%)	2 (8%)	14 (25%)
Alcohol use disorder	11 (9%)	2 (8%)	4 (7%)
Substance use disorder	2 (2%)	0	1 (2%)
Obesity	68 (56%)	15 (60%)	32 (56%)
Diabetic complications	4 (3%)	0	3 (5%)
Trimester of first prenatal visit			
1st	92 (79%)	22 (88%)	42 (74%)
2nd	19 (16%)	3 (12%)	11 (19%)
3rd	6 (5%)	0	4 (7%)

HbA1c at pregnancy baseline was the only patient characteristic found to be a significant predictor of the clinical outcome in univariate analyses (OR = 1.821, 95% CI = 1.184–2.801, *P* = 0.006). This held true when the analysis was repeated excluding the 22 pregnancies with no post-pregnancy HbA1c data (*not presented*). Of the baseline comorbidities explored, none were statistically significant. Because of this, stepwise fitting of a multivariate regression model was not pursued (Table 2).

**Table 2:** Univariate logistic regression analyses for identifying predictors of unfavorable outcome

Covariate	OR (95% CI)	*P*-value
Age	0.950 (0.875–1.030)	0.215
Medicaid status	1.159 (0.266–5.058)	0.844
HbA1c	1.821 (1.184–2.801)	0.006
BMI	0.968 (0.887–1.057)	0.469
Hypertension	1.031 (0.344–3.092)	0.957
Hyperlipidemia	1.093 (0.386–3.096)	0.867
Cardiovascular disease	0.639 (0.100–4.082)	0.636
Depression	3.744 (0.782–17.920)	0.098
Alcohol use disorder	0.868 (0.148–5.078)	0.875
Substance use disorder	–	–
Obesity	0.853 (0.328–2.220)	0.745
Diabetic complications	–	–
Trimester of first prenatal visit	2.578 (0.784–8.483)	0.119

### HbA1c trends

We explored trends in HbA1c for all pregnancies, comparing values from pre- and post-pregnancy periods as defined above. Fifty-nine pregnancies had a set of HbA1c data points from pre-, during, and post-pregnancy. Nearly three-fifths of women (*n* = 35) had glycemic control before their pregnancy, and all but two of these kept their HbA1c < 8% during pregnancy. Thirteen women switched to uncontrolled after pregnancy (defined as any HbA1c reading ≥8%), so that only 57% (*n* = 20) of those who entered pregnancy well-controlled were able to maintain glycemic control in the 2 years post-pregnancy.

**Fig. 1:**
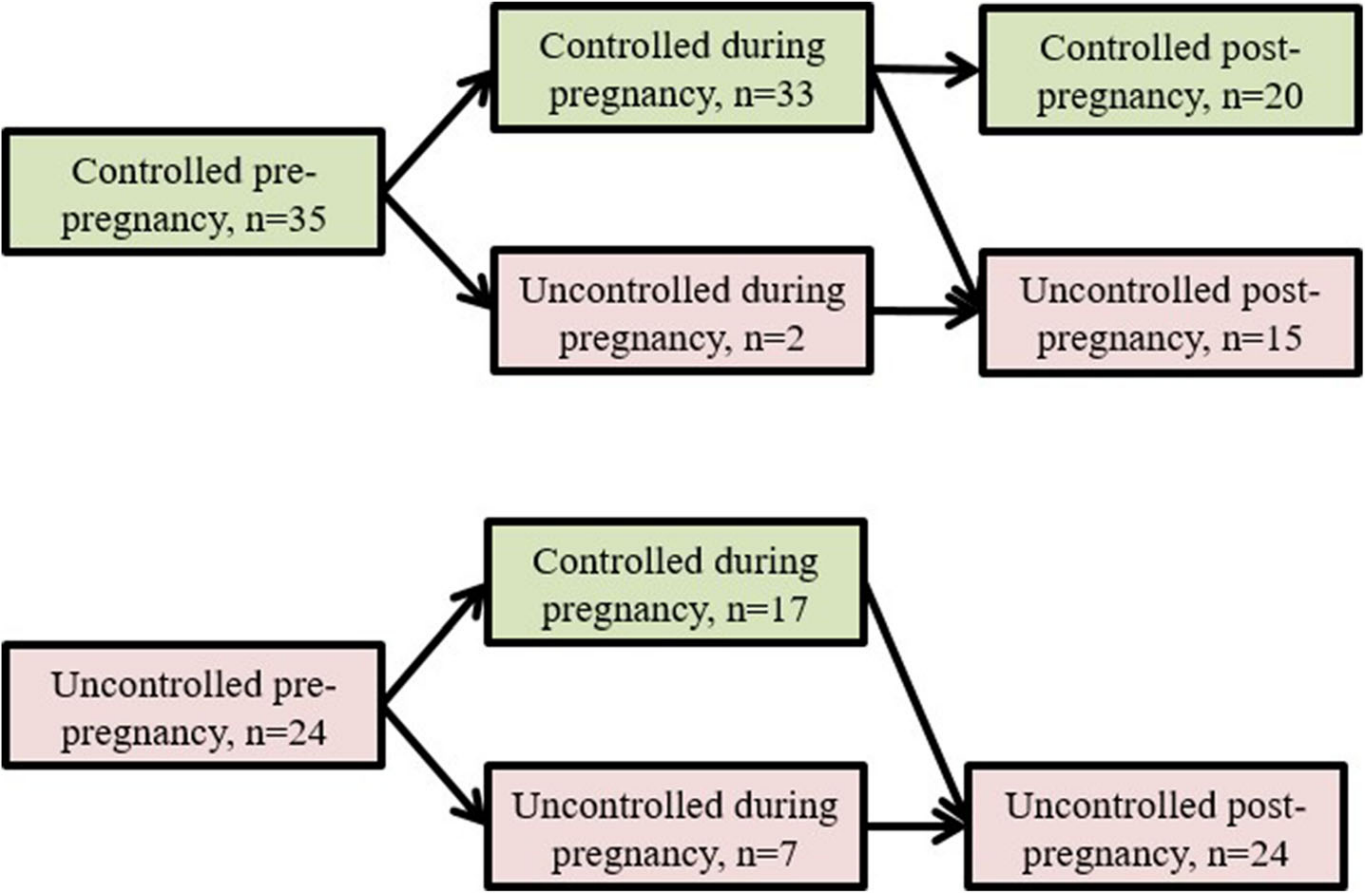
“Trends in HbA1c control stratified by status pre-pregnancy”

For the two-fifths of women (*n* = 24) who had uncontrolled diabetes before pregnancy, 71% (*n* = 17) were able to keep their HbA1c < 8% during pregnancy. However, all of these subsequently became uncontrolled in the post-pregnancy period (Fig. 1).

### Healthcare utilization after pregnancy

We examined care utilization after pregnancies with a known end date (*n* = 82). Seventy-nine women (96%) had an encounter of some sort within the service unit. These were all within the postpartum period, with a median of 7 days and max of 49 days postpartum. Forty-three women (52%) were seen by obstetrics or gynecology, and 10 women (12%) received care at a primary care clinic (internal medicine and family practice, no pediatrics visits were found). Overall, 50 women (61%) received obstetrics/gynecology and/or primary care in the postpartum period. Thirty-two women had some other healthcare encounter in the postpartum period (such as pharmacy, radiology, or various ancillary departments) but no visit to either obstetrics/gynecology or primary care.

Looking beyond the immediate postpartum period, 71 women received some form of care from obstetrics, gynecology, or primary care within the IHS service unit in the 2 years following their partum date. The median time to care was 23 days, and the maximum was 577 days post-pregnancy. Forty women (49%) accessed a primary care provider within 2 years.

**Fig. 2:**
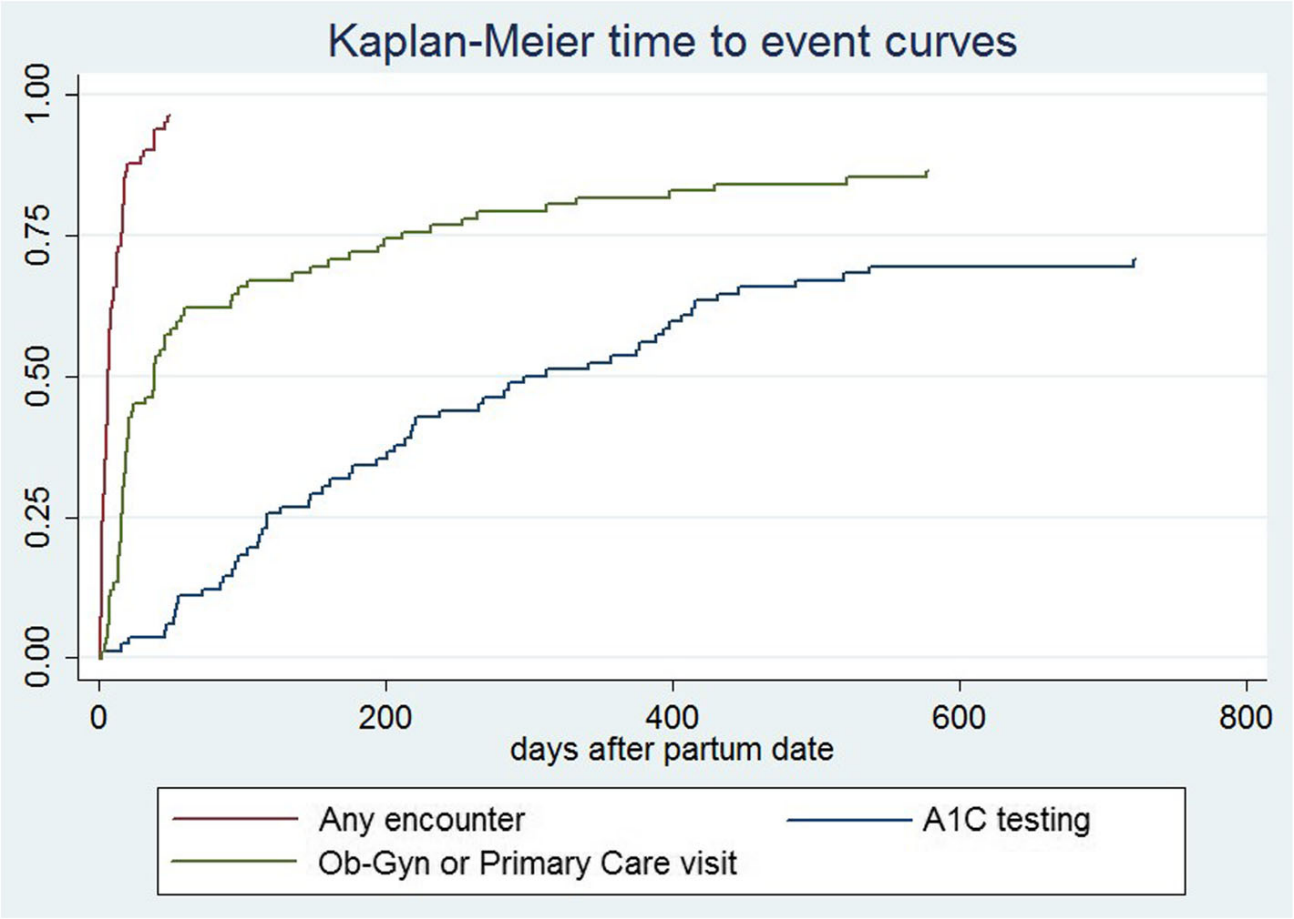
“Time to key clinical events post-pregnancy”

While nearly all women received healthcare services in the first 8 weeks after delivery, the first HbA1c test after pregnancy generally occurred much later, with the median time-to-first HbA1c being 197.5 days. Twenty-four women (29%) had no HbA1c recorded within 2 years (Fig. 2).

## Discussion

This analysis explores diabetes outcomes among women living with diabetes mellitus who received IHS care on Navajo Nation and had at least one pregnancy. Among women whose pregnancies could be tracked until completion, only 30% achieved adequate diabetes control after pregnancy, as defined by HbA1c measures < 8% during the 2 years post-pregnancy. None of the patient comorbidities assessed were predictive of outcome in our univariate analyses, although small study size may have prevented several (depression, substance use disorder, and complicated diabetes) from reaching statistical significance. Depression and diabetes in pregnancy have been studied extensively without clear consensus about their associative relationship, and our study again underscores a need for specific research into this area [24]. The link between these and alcohol/substance abuse, which together have been described as a comorbid “triad”, are certainly plausible from a socioecological and biological perspective [25]. The only individual characteristic which predicted an unfavorable outcome was HbA1c value at baseline. This finding—that a woman’s level of diabetes management pre-pregnancy is statistically associated with diabetes outcome post-pregnancy—is intuitive but important to note in our unique population.

The trends in HbA1c trajectory for our cohort of pregnant women likely reflects success of the Diabetes In Pregnancy team at the healthcare facility. Approximately 85% of women were able to maintain their HbA1c < 8% throughout pregnancy; moreover, 71% of women (17 in 24) who entered pregnancy with uncontrolled diabetes were able to achieve this. On the other hand, an ongoing challenge appears to be maintaining diabetes control post-pregnancy, after women leave the care of the diabetes in pregnancy team. Sixty-percent of the women (30 of 50) who were controlled during pregnancy had a subsequent uncontrolled HbA1c within 2 years, including all the women who entered pregnancy uncontrolled. Taken together, our findings—that women are able to achieve control when supported and highly motivated during pregnancy, but then trend back toward uncontrolled diabetes due to competing priorities and/or loss to follow-up—echo the conclusions of other studies conducted on pre-existing diabetes in pregnancy [26–28].

Impressively, we found that 96% of women were seen within the studied IHS service unit during the eight-week postpartum period. Only 61% of women saw an ob-gyn or primary care provider during this important time, whereas the others accessed other ancillary services. Timely follow-up is an important part of high quality postpartum care and management of diabetes in pregnancy, as described in recommendations from both the American College of Obstetrics and Gynecology and the American Diabetes Association [19, 29]. Care must be comprehensive to tend to women’s physical and psychosocial wellbeing, provide anticipatory guidance, as well as assess diabetes status. Patient perspectives have been provided by others on the barriers and facilitators to seeking postpartum diabetes care, and insights specific to IHS-served populations and Navajo Nation are needed [30].

While it is encouraging that women are interacting with the healthcare system during the postpartum period, we found that less than half of women had at least one primary care encounter within the 2 years after having their child. The transition from pregnancy into routine primary care is marked by challenges for women, including the stress of a newborn at home and long wait times to see a primary care provider. The interconception care period, centering around a woman and family from the birth of one child to the next, could be a focal point for strengthened service delivery in the future [31]. Interconception care strategies could include offering integrated clinical services for the mother and child, empanelment to a primary care provider as part of prenatal care, as well as community outreach to women who are lost to follow-up. Our results also highlight women’s self-efficacy in successfully managing diabetes during pregnancy (even among those who were uncontrolled prior to pregnancy) as a potential empowering motivation for women to continue this trajectory after delivery.

Among our cohort of women with diabetes mellitus, we observed that time to HbA1c testing occurred a median of 197.5 days after delivery, and that no test was performed in the 2 years after delivery for 29% of women. It is important to note that guidelines do not recommend HbA1c testing in the immediate postpartum period, since high red blood cell turnover affects the sensitivity of hemoglobin-based screening. However, testing is recommended at least twice a year for all persons with diabetes and more frequently for those with recently changed treatment or uncontrolled disease (which describes almost all women in the diabetes in pregnancy program) [21]. We found that achieving this measure of routine diabetes management and “true” primary care was the slowest of our three endpoints.

The fact that women are interacting with the system suggests that there are missed opportunities to get women into primary care and continue to provide diabetes care, including HbA1c testing. The pediatrics department at our study site has bundled newborn visits, which are well-attended, with basic screening/counseling for mothers to try and connect them back into care. Numerous other examples of interventions to improve postpartum diabetes screening have been studied, and proactive systems-based approaches have been shown to improve follow-up [32].

This analysis has several limitations. We did not assess pregnancy outcomes (e.g. complications of pregnancy and delivery) nor were we able to evaluate outcomes among children, based on available data. Missing data was also a limitation: nearly one-third of pregnancies (*n* = 40) were missing outcomes and others lacked a baseline HbA1c (*n* = 32) and/or BMI (*n* = 51), introducing the possibility of selection bias. Comparison of the pregnancies with known and missing outcomes shows a statistically higher baseline HbA1c in the latter. This, along with the fact that an absence of HbA1c and BMI measurements is itself an indicator of suboptimal care, suggests that our complete case analyses omit women with poorer diabetes control and engagement in care.

Given our single-site design, we speculate another explanation for missing data is that some women presented at our facility for partial care, but received additional care at other facilities. Women with high risk pregnancies or those with Medicaid or private insurance, for instance, might be more likely to have access care elsewhere. Findings must therefore be interpreted through the lens of services provided at a single facility, which is part of a larger network of care.

## Conclusion

Native Americans living on reservations experience a range of health disparities, among which diabetes morbidity and mortality is prominent. The Indian Health Service is an important resource for this population, and IHS providers continue to look for ways to advance the mission of providing high-quality care and reducing inequity [2, 33]. This analysis focuses on the issue of diabetes in pregnancy among women seen in an IHS facility on Navajo Nation. Our results suggest that many women do not sustain diabetes control and continuity of care into the post-pregnancy period. On the other hand, our findings suggest that local efforts to address diabetes in pregnancy are having a positive impact on ensuring that women have optimal glycemic control during pregnancy. This work highlights the importance of implementing strategies to ensure that women successfully transition to ongoing primary care and continued glycemic control, and offers a direction forward through future collaborative efforts between the community and health professionals.

## Abbreviations

BMI: Body mass index
COPE: Community Outreach and Patient Empowerment
HbA1c: Hemoglobin A1c
IHS: Indian Health Service
