# Correction to: Glycemic control and healthcare utilization following pregnancy among women with pre-existing diabetes in Navajo Nation

**DOI:** 10.1186/s12913-019-4568-1

**Published:** 2019-10-22

**Authors:** Julius Ho, Karen Bachman-Carter, Shelley Thorkelson, Kristi Anderson, Jennifer Jaggi, Chris Brown, Adrianne Katrina Nelson, Cameron Curley, Caroline King, Sid Atwood, Sonya Shin

**Affiliations:** 1 Department of Medicine, Johns Hopkins School of Medicine, 1800 Orleans St, Baltimore, MD 21298 USA; 2 Northern Navajo Medical Center, Indian Health Service, Shiprock, NM USA; 3 Gallup Indian Medical Center, Indian Health Service, Gallup, NM USA; 4 Division of Global Health Equity, Brigham and Women’s Hospital, 210 East Aztec Avenue, Gallup, NM 87301 USA; 5 Community Outreach and Patient Empowerment, 210 East Aztec Avenue, Gallup, NM 87301 USA; 6 Department of Global Health and Social Medicine, Harvard Medical School, 641 Huntington Ave., Boston, MA 02115 USA

**Correction to: BMC Health Serv Res (2018) 18:629**

**https://doi.org/10.1186/s12913-018-3434-x**

In the original publication of this article [1], an author’s name needs to be revised from Katrina Nelson to Adrianne Katrina Nelson, and this author's affiliation address should be "641 Huntington Ave., Boston, MA 02115".  The origial article has been corrected for the author's name.
